# RNA Sequencing of *Populus x canadensis* Roots Identifies Key Molecular Mechanisms Underlying Physiological Adaption to Excess Zinc

**DOI:** 10.1371/journal.pone.0117571

**Published:** 2015-02-11

**Authors:** Andrea Ariani, Daniela Di Baccio, Stefania Romeo, Lara Lombardi, Andrea Andreucci, Alexander Lux, David Stephen Horner, Luca Sebastiani

**Affiliations:** 1 BioLabs-Institute of Life Sciences, Scuola Superiore Sant'Anna, I-56127 Pisa, Italy; 2 Department of Biology, Università degli Studi di Pisa, I-56126 Pisa, Italy; 3 Department of Plant Physiology, Faculty of Natural Science, Comenius University in Bratislava, Bratislava, Slovakia; 4 Institute of Chemistry, Slovak Academy of Sciences, Bratislava, Slovakia; 5 Department of Biosciences, Università degli Studi di Milano, Milano, Italy; Austrian Federal Research Centre for Forests BFW, AUSTRIA

## Abstract

*Populus x canadensis* clone I-214 exhibits a general indicator phenotype in response to excess Zn, and a higher metal uptake in roots than in shoots with a reduced translocation to aerial parts under hydroponic conditions. This physiological adaptation seems mainly regulated by roots, although the molecular mechanisms that underlie these processes are still poorly understood. Here, differential expression analysis using RNA-sequencing technology was used to identify the molecular mechanisms involved in the response to excess Zn in root. In order to maximize specificity of detection of differentially expressed (DE) genes, we consider the intersection of genes identified by three distinct statistical approaches (61 up- and 19 down-regulated) and validate them by RT-qPCR, yielding an agreement of 93% between the two experimental techniques. Gene Ontology (GO) terms related to oxidation-reduction processes, transport and cellular iron ion homeostasis were enriched among DE genes, highlighting the importance of metal homeostasis in adaptation to excess Zn by *P. x canadensis* clone I-214. We identified the up-regulation of two *Populus* metal transporters (*ZIP2* and *NRAMP1*) probably involved in metal uptake, and the down-regulation of a *NAS4* gene involved in metal translocation. We identified also four Fe-homeostasis transcription factors (two *bHLH38* genes, *FIT* and *BTS*) that were differentially expressed, probably for reducing Zn-induced Fe-deficiency. In particular, we suggest that the down-regulation of *FIT* transcription factor could be a mechanism to cope with Zn-induced Fe-deficiency in *Populus*. These results provide insight into the molecular mechanisms involved in adaption to excess Zn in *Populus* spp., but could also constitute a starting point for the identification and characterization of molecular markers or biotechnological targets for possible improvement of phytoremediation performances of poplar trees.

## Introduction

Zinc (Zn) is an essential micronutrient involved in several physiological and metabolic processes as a structural or catalytic co-factor in a large number of enzymes and regulatory proteins [1]. However, Zn excess causes serious defects such as chlorosis and plant growth inhibition [2]. In the recent decades, human industrial activities have contributed to increasing Zn contamination of soils, reaching, in some areas, potentially toxic levels for both plants and animals [3], [4]. Current techniques for removing Zn pollution are expensive and damaging for the environment, emphasizing the need for cost-effective and eco-friendly alternatives such as phytoremediation (the decontamination of soils using plants) [5].

While poplar exhibits lower metal accumulation than herbaceous hyperaccumulator plants [6], [7], several studies have identified the genus *Populus* as suitable for phytoremediation approaches [8–10] due to its great biomass production, high growth rates and deep and developed root systems [11]. Moreover, *Populus* is the internationally accepted model system for physiological and molecular studies in woody plants, in part due to the availability of the complete genome sequence of *P*. *trichocarpa* (Torr. & Gray) [12].

With the advent of high-throughput technologies, such as microarray and Next Generation Sequencing, several large-scale transcriptome analyses have been performed to elucidate the molecular basis of poplar physiological and developmental mechanisms [13], [14], as well as the response of the *Populus* transcriptome to both biotic and abiotic stresses [15–17]. Responses and adaptation to excess Zn and other heavy metals have also been studied in poplar [18]-[22], but little is known of the molecular mechanisms involved in heavy metal homeostasis and tolerance in roots [21], [23], [24]. Plant root system is directly involved in interactions with the environment and responsible for nutrient homeostasis and water uptake [25]. However, despite its importance, this organ is generally poorly characterized at the molecular level in poplar [21], [25]. Physiological studies on *P*. *x canadensis* clone I-214 exposed to excess Zn report a general indicator phenotype for this plant [7], [24], [26]. This clone showed a limited translocation to aerial parts [19], [20], possibly to protect the photosynthetic apparatus from excess Zn. Anatomical studies and X-ray dispersive microanalysis of I-214 roots under excess Zn highlight the differentiation of apoplastic barriers and lignification processes towards the root apex, with higher Zn concentration in cortical tissues [27]. Similar anatomical modifications have been reported in both maize and willow plants in response to excess Cd [28]. Such modifications seem to represent mechanisms for controlling or reducing metal translocation to the aerial parts by developing physical barriers at the root level.

Whole transcriptome sequencing (RNA-seq) provides unprecedented levels of accuracy and sensitivity in differential gene expression than alternative high-throughput technologies, such as microarray [29]. Although RNA-seq possesses clear advantages, this technology poses significant informatics challenges due to the production of millions of short sequences which are meaningless until assembled into or assigned to full transcripts whose expression can be quantified [29]. In addition, differentially expression analysis by RNA-seq could be challenging in plant species with a highly redundant genome, and a high degree of intra- and inter-specific variation, such as *Populus* [30].

*Populus x canadensis* clone I-214 was identified in a screen of poplar clones and has been characterized for response to excess Zn at the physiological, ultra-structural, anatomical and molecular level [18], [19], [20], [27]. All these studies suggest 1mM Zn as a sub-lethal but symptomatic concentration for this poplar clone in hydroponic conditions. Few information regarding the molecular mechanisms underlying metal homeostasis and stress-response in roots is available to date. Here we used RNA-seq to study the response of the root transcriptome to excess Zn. For increasing the specificity of our analysis we consider the most significant DE genes identified by three statistical algorithms. The expression of these genes was measured by RT-qPCR, and only the genes whose differential expression was confirmed by RT-qPCR were considered for further biological interpretations. Results are considered in the light of growth parameters and patterns of Zn accumulation/distribution and compared to previous data for poplar leaves. The main focus is the variation in root transcriptome and its correlations with physiological responses. This study provides information about molecular mechanisms underlying regulation of homeostasis, tolerance and adaptation to excess Zn in poplar. To our knowledge, this is also the first report of differentially expression analysis in response to excess Zn in poplar roots using RNA-sequencing approach.

## Materials and Methods

### Plant material and growth conditions

Woody cuttings of the hybrid poplar *Populus x canadensis* clone I-214 were provided by CRA—Unità di Ricerca per le Produzioni Legnose Fuori Foresta, Casale Monferrato (Alessandria, Italy).

After rooting, woody cuttings were transplanted to plastic pots containing Agrileca clay (3–8 mm diameter, Laterlite, Milano, Italy) and cultivated in a controlled climate chamber in a floating hydroponic system for 1 month. The pots floated in rectangular plastic tanks filled with 5 L of a modified full-strength Hoagland's nutrient solution (renewed every 7 days) was applied at pH 6.2 [20]. The iron was supplied as Fe-tartrate instead of Fe-EDTA for avoiding possible Zn chelation by EDTA. Each tank contained 5 pots/plants and for the experiment, 4 tanks were set up. Nutrient solution aeration (250 L h^-1^) was provided by aquarium pumps and the growth chamber conditions were set as follow: 23–18°C day-night temperature, 65–70% relative humidity and a photoperiod of 16/8h light/dark at a maximum photon flux density of 400 μmol m^-2^ s^-1^ (photosynthetically active radiation) supplied by fluorescent lights. After 1 month of acclimation to hydroponic conditions, 13 plants were chosen and maintained by a unique stem; immediately before the application of Zn treatments, three plants were used to determine the initial (*t0*, time zero) dry weight (DW) partitioning among different plant organs (leaves, stem, roots and cutting). The 10 remaining plants were randomly assigned to the following two Zn treatments (n = 5): (i) basic Hoagland's solution containing 1 μM Zn (corresponding to 0.065 ppm, i. e. the control), 5 plants; (ii) basic Hoagland's solution containing 1 mM Zn (65 ppm, i. e. the treatment), 5 plants. Zn was supplied as Zn(NO_3_)_2_·6H_2_O. For each Zn treatment the plant were grown in a single tank.

The plants were subjected to the above mentioned Zn treatments for 3 weeks (21 days) under the nutritional and environmental conditions described. At the end of this period visible stress symptoms started to become apparent as slight chlorosis on young leaves in plants subjected to 1mM Zn compared to control as previously described [19], but any modifications was observed in roots. Each analysis was performed at the end of Zn treatments. At the end of the experiment (3 weeks), plants were harvested and the different plant parts (leaves, stems, roots and woody cuttings) were divided for downstream biomass and molecular analysis.

### Growth parameters and Zn content analyses

For biomass and Zn content determinations, plant organs (leaves, stems, roots and woody cuttings) from five plants (n = 5) in each Zn treatment were sampled separately and after the collection of few mg of fresh weight from leaves and roots for DNA or RNA isolation, all the remaining material was oven dried at 60°C until their weights remained constant. Leaf area was measured on all the fresh leaves of each plant immediately after cutting from the plants using scanner and image analysis software (Scion Image, release 4.0.2, Scion Corporation, Frederick, MD, USA). The relative growth rate (RGR) and specific leaf area (SLA) were determined as previously described [19]. For each control and Zn treated plant the total dry samples of leaves, stem and roots were ground separately into a fine powder in an analytical mill (IKA-werke GmbH & Co. KG, Staufen, Germany) for subsequent Zn determinations. Zinc was quantified in different plant organs after digestion in concentrated nitric acid (HNO3) by atomic absorption spectrophotometry (model 373; PerkinElmer, Norwalk, CT, USA), as previously described [31]. For each plant organ the Zn concentration (μg g DW^-1^) and Zn content (mg) were shown.

### Genomic DNA isolation and re-sequencing

Genomic DNA isolation was performed from frozen leaves using DNeasy plant mini kit (Qiagen, Hilden, Germany) according to manufacturer's protocol. Sequencing libraries were constructed using the TruSeq DNA sample kit from Illumina (Illumina, Inc., CA, USA) according to manufacturer's instructions. DNA-seq libraries were loaded into a single lane of a flow cell and sequenced at 2x101 bp set-up with a Genome Analyzer IIx sequencer by IGA Technology Services, Udine, Italy.

Genomic sequencing generates 21831070 pairs of 100 bp genomic DNA reads (43662140 reads, with a theoretical insert size of 600 bp) for the *P*. *x canadensis* clone I-214 genome. Analysis of quality score distributions with fastx-toolkit (http://hannonlab.cshl.edu/fastx_toolkit/) suggested that error rates increased sharply after the 70^th^ base of both the forward and reverse reads. Accordingly all reads were trimmed to 70 bases in length before mapping to the *P*. *trichocarpa* genome with SOAP2 [32]. The alignment parameters were set up as follow: no constraint on DNA strand mapping positions and unique best mapping solutions with 5 or less mismatches. After the alignment only reads that map within 100 bases of the expected insert size were considered for downstream analysis (38092096 individual mapped reads). Custom scripts (available upon request) were used to reconcile mismatches in mapped reads to the coordinates of annotated *P*. *trichocarpa* genes, to assist in the design of primers for RT-qPCR experiments. Raw genomic reads are available at NCBI sequence read archive (http://www.ncbi.nlm.nih.gov/sra, accession number: SRX247021).

### Root RNA isolation and differential expression analysis

Samples obtained from the whole root apparatus of five plants (n = 5) in each Zn treatment were frozen in liquid nitrogen, grinded and stored at −80°C. Total mRNA was extracted from 80–100 mg of root samples using the RNeasy Plant Mini Kit (Qiagen, Hilden, Germany), according to the manufacturer's protocol. Genomic DNA traces were removed using the RNase-free DNase set (Qiagen, Hilden, Germany). RNA quality and concentration were evaluated with the Experion automated station using the Experion RNA StdSens kit (Biorad, Berkely, CA, USA) according to manufacturer's protocol. For each Zn treatment, total root RNA was pooled by mixing equal amounts (as μg of total extracted RNA) from the five independent plant extractions of root samples. Total RNA was concentrated using the RNeasy MinElute Cleanup Kit (Qiagen, Hilden, Germany). The two (1 μM Zn, control, and 1 mM Zn) pooled concentrated RNA samples were used for RNA sequencing. 2μg of total mRNA was used for preparing the sequencing libraries.

Sequencing libraries, enriched in PolyA^+^ RNA fraction, were constructed using the TruSeq RNA sample prep kit from Illumina (Illumina, Inc., CA, USA) according to manufacturer's instruction. RNA-seq libraries were loaded into a single lane of flow cell and sequenced for 44 cycles with a Genome Analyzer IIx sequencer by IGA Technology Services, Udine, Italy.

RNA sequencing generated 23168921 and 20331174 reads of 43 bp length from the control and Zn-treated libraries, respectively. Analysis of quality score distributions with fastx-toolkit (http://hannonlab.cshl.edu/fastx_toolkit/) did not indicate a significant reduction of sequence quality towards the ends of the reads. Reads were mapped to the *Populus trichocarpa* (v2.2) transcriptome downloaded from Phytozome (http://www.phytozome.net/) with SOAP2 [32], allowing a maximum of 5 mismatches and accepting only unique best mappings. To each transcript was assigned a number of reads by counting the number of reads that map to it. Where appropriate, reads per transcript were normalized as reads per transcript per million mapped reads for the total number of uniquely mapped reads (14311904 and 12096737 for control and Zn treatments respectively).

Transcript read counts were analyzed for differential expression using three different strategies. First, for each transcript a χ^2^ test based on a 2x2 contingency table was performed using custom Python scripts (available upon request). Secondly, the DESeq [33] biocondutor package was employed using default settings and with negative binomial distribution parameters estimated using the “pool = TRUE” option, as biological replicates were not available. *P* values, corrected for multiple testing, were saved for differential expression of each transcript. Finally, we employed the ASC methodology [34], to calculate Bayesian posterior probabilities of differential expression. Subsets of candidate DE genes were extracted accordingly to two differently stringency analyses. In the first, more stringent, dataset we included the genes with a DESeq adjusted *P* value ≤ 0.05, a posterior probability greater than 0.95 and a ≥ 2-fold difference of expression, and a Bonferroni corrected *P* value lower than 0.1 for the χ^2^ test. This first approach, even though probably reducing the identification of false positives, could also determine the loss of biological signals from the dataset. For this reason we identified also a second, less stringent, dataset including the 500 most significant candidate up-regulated and down-regulated genes identified by each of DESeq and the χ^2^ test (1000 genes for each algorithm), with the 1000 genes with the highest posterior probability and ≥ 2-fold difference (up-regulated or down-regulated) in expression levels identified by ASC (506).

Raw mRNA sequencing reads are available at NCBI sequence read archive (http://www.ncbi.nlm.nih.gov/sra, accession number: SRX246820, SRX247014).

In order to confirm the RNA-seq data transcriptomic screening, the up-regulated and down-regulated genes previously identified were deeply studied by RT-qPCR analysis performed on biological replicates. For RT-qPCR analysis 1 μg of total root RNA independently extracted from three (n = 3) of the five sampled poplar plants in each Zn treatment. Total RNA was reverse transcribed using the High Capacity cDNA Reverse Transcription Kit (Applied Biosystems) according to manufacturer's protocol. RT-qPCR reactions were performed using SsoFast EvaGreen supermix (Biorad, Berkeley, CA, USA) on Eco Real-Time PCR System (Illumina, Inc., CA, USA), applying an annealing/extension step for 20 sec at 55°C for 40 cycles. The qPCR reactions were performed in a total volume of 15 μl, with a final primer concentration of 0.3 μM and 0.5 ng of cDNA per reaction. For every biological replicates, 3 distinct technical replicates of the qPCR reaction were performed and averaged. Significance of differential expression was determined using the mean Fold Change (FC) in a t-test comparison implemented in R (www.r-project.org). The melting curves, obtained adding the dissociation step at the end of the amplification cycles, were checked with default parameter for each RT-qPCR reaction to evaluate the presence of multiple amplification fragments and/or mRNA isoforms. Several reference genes [35] were tested for RT-qPCR and EF1β has been selected as the more suitable reference gene for our experimental condition. Correlation analysis of log_2_ FC of the DE genes between bioinformatic prediction and RT-qPCR assay was performed with R.

### Comparison of the DE genes between roots and leaves

The genes identified as DE from a previous microarray analysis on the same poplar clone (*Populus* x *canadensis*, I-214) subjected to the same Zn treatments [20] were compared with those identified in this study. The annotation file of *P*. *trichocarpa* array was downloaded from http://www.affymetrix.com/analysis/index.affx and used for converting the affymetrix gene ID to the *P*. *trichocarpa v 2*.*2* gene ID. The genes DE in leaf were compared with those that showed a similar and significant trend in differential expression also in root (i. e. up- or down-regulated in both root and leaves). In order to find the common DE genes in both organs, the leaf DE genes were compared with the root DE genes of both the first, more stringent, and the second, less stringent, datasets.

### Gene Ontology Enrichment Analysis

The genes identified as DE in response to excess Zn in root of *P*. *x canadensis* clone I-214 were manually annotated using blast2go software [36], and subsequently analyzed with the same software for evaluating the most statistically represented GO terms in our dataset. The full *P*. *trichocarpa* transcriptome GO terms were downloaded from agriGO (http://bioinfo.cau.cn/agriGO/), and used as background genome annotation for evaluating the over-representation of specific GO terms in our data set (GO enrichment analysis).

Gene Ontology enrichment analysis was performed using Fisher exact test of blast2go outputs on the first, more stringent, dataset, by analyzing the up-regulated and down-regulated gene clusters together and separately, with an adjusted false discovery rate (FDR) corrected *P* value cut-off of 0.05. The same analyses were performed on the second, less stringent dataset on the entire set of DE genes, with (FDR) corrected *P* value cut-off of 0.005.

## Results

### Growth and Zn concentration and content analyses

Under Zn stress the I-214 leaf biomass and leaf area of 1 mM Zn treated plants were significantly reduced by about 1.4-, and 1.2-fold compared to control (1 μM Zn) plants (3.02 g and 2.15 g − 1376.6 cm^2^ and 1114.9 cm^2^, respectively, Table 1). These modifications decreased the total biomass production (-23%) and the shoot to root ratio (-15%) of Zn treated plants. On the contrary, significant differences in biomass allocation in stem, root and woody cutting, the number of leaves, SLA and RGR were not observed. Excess Zn caused a statistical significant increase (from 5- to 83-fold higher) in both the concentration and content of this metal in all plant parts, in comparison to control condition. The higher Zn concentration was observed in Zn treated roots, with a ~70 fold increase compared to control, but also stem and leaf showed increased Zn accumulation of about 6 and 8 fold, respectively (Table 1). At Zn 1 mM the enhancement of Zn concentration in the stem was similar to that registered for the metal content in the same organ (6- and 5-fold higher, respectively). On the other hand, in the treated leaves the 8-fold increase of Zn concentration corresponded to a 5.6- fold increase in Zn content, and in the roots the 70-fold increase of Zn concentration corresponded to a 83-fold increase when compared to control. The total Zn accumulation in treated plants was more than 10 time higher than in the controls (Table 1).

**Table 1:** Growth and Zn content analyses.

	Zn treatment		
Parameter	Control	1 mM	*P*-values
Root (g)	0.44±0.06	0.39±0.1	0.39
Stem (g)	1.57±0.15	1.34±0.37	0.24
Leaf (g)	3.02±0.4	2.15±0.47	0.02 (*)
Woody cutting (g)	11.43±4.17	16.81±5.84	0.15
Total biomass (g)	5.03±0.57	3.88±0.92	0.05 (*)
			
RGR (day^-1^)	0.083±0.005	0.070±0.012	0.06
Shoot/Root	10.5±1.2	8.9±0.3	0.04 (*)
Leaf Number	23.4±1.1	23.5±2.6	0.94
Leaf Area (cm^2^)	1376.6±42.4	1114.9±6.1	0.03 (*)
SLA (m^2^ kg^-1^)	42.1±3.6	44.2±1.1	0.55
			
Root Zn (μg g^-1^)	55.1±32.5	3808.8±793.9	0.002 (**)
Root Zn (μg)	24.8±16.5	2057.6±1167.2	0.04 (*)
Stem Zn (μg g^-1^)	39.6±5.5	235.5±19.3	0.0001 (***)
Stem Zn (μg)	62.6±13.7	312.4±82.0	0.0003 (***)
Leaf Zn (μg g^-1^)	105.0±19.4	842.3±43.4	0.0001 (***)
Leaf Zn (μg)	322.4±96.4	1808.0±401.1	0.0001 (***)
Total Zn (μg)	401.7±121.4	4136.9±1077.5	0.0001 (***)

Growth parameters and Zn concentration and content in roots, stem and leaves of *Populus x canadensis* clone I-214 subjected to 1 μM (control) and 1 mM Zn treatments. Data reported are the means ± s.d. (n = 5). Results of student t-test are shown. DW = dry weight. Shoot/root was calculated using the sum of leaf and stem DW/root DW. RGR was calculated using the root, stem and leaf DW determined at time *t0* and after 3-week Zn treatments. RGR = Relative Growth Rate; SLA = Specific Leaf Area. The total biomass, the RGR and shoot/root ratio were calculated excluding woody cuttings.

* = P ≤ 0.05

** = P ≤ 0.01

*** = P ≤ 0.001

### Identification of DE genes

**Figure 1:**
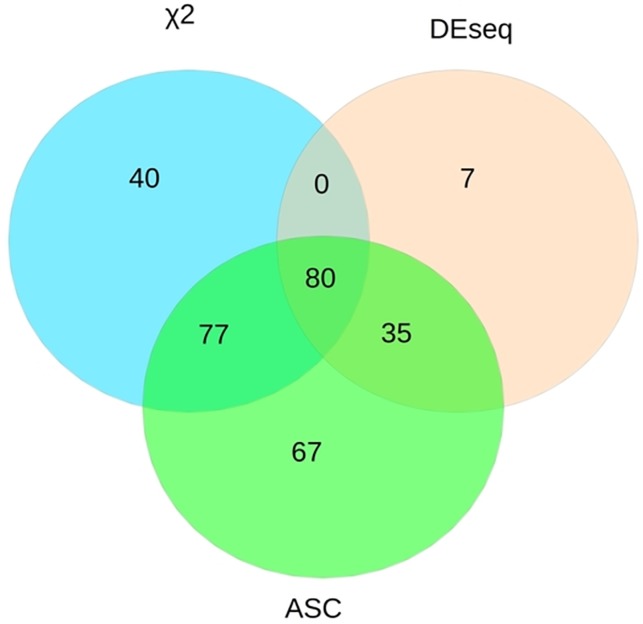
Identified differentially expressed genes with different statistical approaches. Venn diagram showing the genes identified as DE by the three different statistical methods (DEseq, ASC and χ2) and the intersection among them.

Reads were mapped to the *P*. *trichocarpa* reference genome sequence allowing only unambiguous best matches and read counts for annotated genes collated. Three distinct statistical approaches to the identification of DE genes were employed, and the identified genes were divided into two datasets accordingly to different stringency parameters. In the first, more stringent, dataset the DESeq package identified 88 up-regulated and 34 down-regulated genes, the ASC algorithm 163 up-regulated and 96 down-regulated genes, and the *χ* ^*2*^ 131 up-regulated and 66 down-regulated genes. The intersections of predictions for the three methods are shown as Venn diagrams in Fig. 1 and indicate substantial overlap of results among methods, with DESeq being the most conservative method. Among 80 genes identified as significantly DE by all the three statistical approaches, 61 genes were up-regulated (76%) and 19 down-regulated (24%). The *P*. *trichocarpa* genes identified as DE in clone I-214 are shown with annotations generated by blast2go, log_2_ FC from RNAseq and RT-qPCR in Table 2. This set of genes was then subjected to validation by RT-qPCR analysis (see below).

**Table 2:** List of the identified DE genes in the first, more stringent, dataset.

Gene ID ^a^	Enriched GO term^b^	Annotation^c^	*Arabidopsis* homologues^d^	Log2 FC RNA-seq	Log2 FC qPCR	P^e^
POPTR_0004s24370	oxidoreductase activity	protein	AT3G61220	2,43	2,41	0
POPTR_0005s10070	oxidoreductase activity	2og-fe oxygenase family protein	AT3G12900	-1,94	-2,4	0
POPTR_0005s21000	oxidoreductase activity	stearoyl-acp desaturase	AT1G43800	2,52	3,68	0
POPTR_0005s26660	oxidoreductase activity	prolyl 4-hydroxylase alpha	AT1G20270	1,72	2,07	0,01
POPTR_0006s10760	oxidoreductase activity	cationic peroxidase 1-like	AT5G05340	1,57	0,94	0,01
POPTR_0006s15610	oxidoreductase activity	1-aminocyclopropane-1-carboxylate oxidase	ATACO1	2,4	3,48	0
POPTR_0007s03890	oxidoreductase activity	protein	ATADH1	2,69	4,38	0
POPTR_0007s03900	oxidoreductase activity	protein	ATADH1	3,02	4,56	0
POPTR_0007s03910	oxidoreductase activity	protein	ATADH1	3,08	4,46	0
POPTR_0009s06740	oxidoreductase activity	cinnamyl alcohol dehydrogenase-like protein	ATCAD7	1,86	1,09	0,01
POPTR_0010s23790	oxidoreductase activity	probable 2-oxoglutarate fe -dependent dioxygenase-like	AT2G36690	2,37	2,49	0,01
POPTR_0011s16560	oxidoreductase activity	protein	AT5G44440	1,87	1,9	0
POPTR_0014s03660	oxidoreductase activity	cytochrome p450-like protein	CYP82C4	-2,22	-2,94	0
POPTR_0015s15430	oxidoreductase activity	squalene monooxygenase-like	SQE1	3,38	4,59	0,01
POPTR_0016s14030	oxidoreductase activity	cationic peroxidase 1-like	AT5G05340	-1,97	-2,18	0
POPTR_0019s05510	oxidoreductase activity	2-aminoethanethiol dioxygenase	AT5G15120	3,42	5,37	0
POPTR_0002s08090	transport	metal transporter	ATNRAMP6	2,59	1,59	0,01
POPTR_0002s08100	transport	metal transporter	ATNRAMP1	2,33	2,23	0,01
POPTR_0005s03840	transport	heavy metal transport detoxification domain-containing protein	AT5G19090	1.65	0.78	0.01
POPTR_0005s20350	transport	metal transporter	ATNRAMP6	-1,92	-1,69	0
POPTR_0008s04860	transport	protein	ATEXO70H2	-1,85	-1,89	0,01
POPTR_0008s08310	transport	metal tolerance protein 10-like	AT1G16310	-2,54	-2,47	0
POPTR_0008s17960	transport	abc transporter c family member 3-like	ATMRP3	3,64	3,02	0
POPTR_0009s03950	transport	zinc transporter	ZIP2	3,8	2,16	0
POPTR_0010s08250	transport	inorganic phosphate	ATPT2	2,17	2,07	0,01
POPTR_0017s11030	transport	abc transporter b family member	AT3G28345	1,75	1,16	0,01
POPTR_0018s00450	transport	protein	AT1G52190	2,1	3,78	0,01
POPTR_0001s02290	NA	tpr domain protein	AT4G17940	2,23	2,58	0
POPTR_0001s17430	NA	protein	AT5G25460	-2,54	-2,18	0
POPTR_0001s24220	NA	transcription factor myb59-like	ATMYB48	2,06	3,07	0,01
POPTR_0001s27110	NA	protein	AT3G08030	-2,22	-2,56	0
POPTR_0001s27680	NA	dna binding	AT2G42280.1	2	1,6	0,01
POPTR_0002s03590	NA	predicted protein [Populus trichocarpa]	AT1G19530	1,78	2,03	0
POPTR_0002s19780	NA	universal stress protein a-like protein	AT3G62550	3,19	3,64	0,01
POPTR_0003s06670	NA	protein tify 10a-like	JAZ6,TIFY11B	-1,78	-0,94	0
POPTR_0004s09920	NA	lob domain-containing protein 41-like	LBD40	2,55	3,89	0
POPTR_0004s18570	NA	protein	UGT74E2	2,28	1,34	0,01
POPTR_0004s20440	NA	nicotianamine synthase	ATNAS4	-3,48	-4,64	0
POPTR_0005s08290	NA	predicted protein [Populus trichocarpa]	NA	1,96	1,61	0,01
POPTR_0005s09890	NA	predicted protein [Populus trichocarpa]	NA	4,09	4,13	0,01
POPTR_0005s15670	NA	lob domain-containing protein 41-like	LBD41	2,41	2,46	0
POPTR_0005s26040	NA	protein	GASA3	2,16	2,46	0,01
POPTR_0005s27980	NA	cp12 domain-containing protein 3	CP12–3	2,12	1,08	0,01
POPTR_0006s03590	NA	transcription factor org2	BHLH038	2,52	2,49	0
POPTR_0006s11870	NA	sequence-specific dna binding transcription factor	AT3G10040	3,5	4,94	0,01
POPTR_0007s00290	NA	predicted protein [Populus trichocarpa]	ARGOS	2,11	2,48	0,01
POPTR_0007s14250	NA	phosphoenolpyruvate carboxykinase	PCK1	-1,79	-4,32	0
POPTR_0008s02280	NA	hippocampus abundant transcript 1	AT2G16980	1,9	1,94	0,01
POPTR_0008s03130	NA	NA	NA	-3,06	-0,64	0
POPTR_0008s06520	NA	transferase family protein	AT2G39980	1,67	1,73	0,01
POPTR_0008s11890	NA	alpha-xylosidase precursor	ATXYL1	-1,89	-1,15	0
POPTR_0008s16430	NA	ethylene receptor	ETR2	1,63	2,11	0,01
POPTR_0008s23260	NA	ap2 erf domain-containing transcription factor	ERF72	1,68	1,73	0,01
POPTR_0009s01100	NA	transcription factor fer-like iron deficiency-induced transcription factor-like	ATBHLH029	-1.75	-2.18	0.00
POPTR_0010s08510	NA	ethylene receptor	ETR2	1,92	2,32	0
POPTR_0010s25680	NA	ests gb	AT1G31130	-2,48	-2,18	0
POPTR_0011s07630	NA	pyruvate decarboxylase	AT5G01320	1,68	2,04	0,01
POPTR_0012s05140	NA	zinc finger	BTS	2,43	2,83	0,03
POPTR_0013s00720	NA	galactinol synthase	AtGolS2	-1,75	-1,79	0
POPTR_0013s01110	NA	universal stress protein	AT3G62550	2,62	3,13	0,01
POPTR_0013s06120	NA	zinc finger	GLIP1	2,07	1,67	0,01
POPTR_0014s06170	NA	beta-expansin 3	ATEXPB2	-1,68	-1,25	0
POPTR_0014s06450	NA	protein	AT3G60780	2,72	2,29	0,01
POPTR_0016s00830	NA	oligopeptide transporter opt family	ATOPT3	2,73	2,66	0,01
POPTR_0016s03680	NA	transcription factor org2	BHLH038	4,04	2,97	0
POPTR_0016s08290	NA	protein aluminum sensitive 3	ALS3	-1,86	-2,18	0
POPTR_0016s10120	NA	glutathione s-transferase protein	ATGSTU8	1,7	1,82	0,01
POPTR_0016s11010	NA	sequence-specific dna binding transcription factor	AT3G10040	3,56	4,15	0
POPTR_0016s12270	NA	protein binding	AT5G01520	2,31	2,6	0,01
POPTR_0017s13320	NA	b12d protein	AT3G29970	3,68	4,68	0
POPTR_0018s03780	NA	flowering promoting factor 1	ATFPF1	1,91	1,22	0,01
POPTR_0018s08240	NA	protein	AT1G52140	2,1	1,74	0
POPTR_0018s13070	NA	ein3-binding f-box protein 1-like	EBF1	1,72	0,98	0,01
POPTR_0018s14270	NA	trans-cinnamate 4-monooxygenase	NA	2,02	2,74	0
POPTR_0018s14280	NA	superkiller viralicidic activity 2-like 2-like	HEN2	1,89	0,03	0,84
POPTR_0019s08630	NA	sorghum bicolor protein targeted either to mitochondria or chloroplast proteins t50848	AT3G56360	1.93	1.37	0.01
POPTR_0019s15220	NA	root phototropism protein 2	RPT2	-2,68	-3,06	0

Annotation of the DE genes, with the differential expression inferred by bioinformatics predictions (log_2_ FC RNA-seq) and RT-qPCR analysis (log_2_ FC RT-qPCR).

^a^Gene ID based on Phytozome v8;

^b^Most significant enriched GO terms to which the genes belong (when available);

^c^Manual annotation with blast2go;

^d^Best *Arabidopsis* TAIR10 hit based on *P*. *trichocarpa* v2 annotation info file (when available the common name for the *Arabidopsis* gene is used);

^e^P-value based on a t-test of relative expression of three biological replicates between control and treatment for RT-qPCR data.

In the second, less stringent, dataset the intersection of the genes with the higher significance or posterior probability in the three different statistical approaches, identify 285 up-regulated and 216 down-regulated genes in total. The list of the candidate genes of this second, less stringent dataset and their normalized log_2_ fold change based on read counts is shown in S1 Table.

### Reconstruction of *Populus x canadensis* clone I-214 gene sequences for PCR primer design

To identify variations between *P*. *trichocarpa* and the hybrid *P*. *x canadensis* clone I-214 genome, and to identify heterozygous sites in the latter, with the aim of generating suitable primers for RT-qPCR to validate candidate DE genes, low coverage (~10x) re-sequencing of the *P*. *x canadensis* clone I-214 genome was performed. Custom scripts (available upon request) were used to identify positions where genome re-sequencing (or RNASeq) data indicated homozygous or heterozygous differences from the reference *P*. *trichocarpa* genome. Primers for validation of differential expression by RT-qPCR were designed upon the codifying regions of the reconstructed genes after RNA-sequencing using the online Primer3 software (http://primer3.ut.ee/), taking care to avoid heterozygous positions and eventual quantification artifacts resulting from differential expression of alleles. Moreover, they have been designed The sequences of the primers used for RT-qPCR validation are shown in S2 Table.

### Validation by RT-qPCR

**Figure 2:**
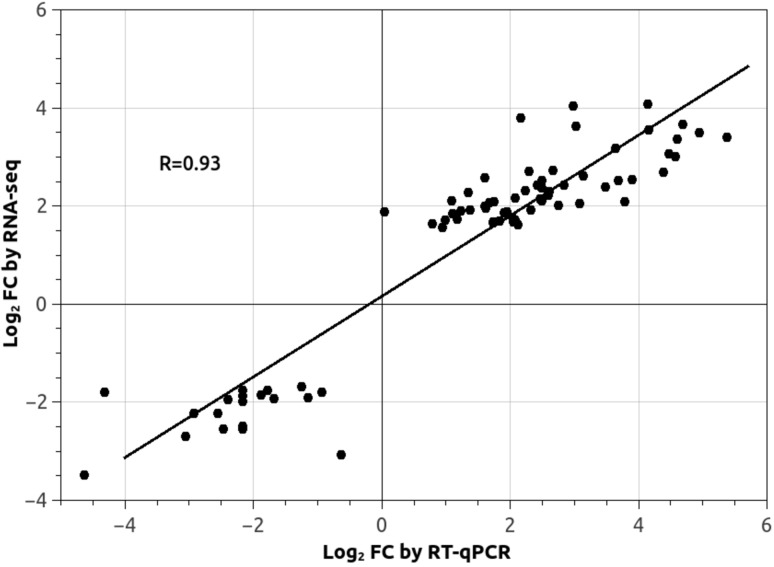
Correlation between the relative quantification of RNA-seq and RT-qCR results. Correlation between differential expression inferred by bioinformatics predictions (log_2_ FC by RNA-seq) and RT-qPCR analysis (log_2_ FC by RT-qPCR). The value represents the log_2_ of the relative fold change (FC) between control and 1 mM Zn treatment. Regression line and Pearson' s correlation coefficient are shown.

Validation of differential expression of genes identified from RNA-seq data in the first, more stringent, dataset was performed using RT-qPCR. Of the 80 genes predicted from RNA sequencing analysis, it was not possible to confidently reconstruct the genomic sequence of three. For all of the other genes (77), RT-qPCR results confirmed the direction of differential expression identified from reads count. T-test comparison for differential expression of RT-qPCR between control and treatment is significant (*P* < 0.05) for all the genes tested, except for POPTR_0018s14280.1 (*P* = 0.84). A strong correlation between the log_2_ fold change of the two methods was observed (Pearson correlation R = 0.93, *P* < 2.2e^-16^) (Fig. 2). The list of the *P*. *trichocarpa* ortholog genes identified as DE with the log_2_ fold change of the two quantification methods are shown in Table 2.

### Comparison of the DE genes between roots and leaves

The comparison between the genes identified in roots in this study and those identified in leaves [20] highlights low overlap of DE genes suggesting a different molecular responses between these organs. Only three genes from the stringent root dataset showed similar expression changes in leaves of Zn stressed plants (1 down- and 2 up-regulated) while 22 of the genes in the less stringent dataset (16 down- and 6 up-regulated genes) were shared with leaf (see Materials and Methods). The list and the annotation of the genes identified as DE from both microarray in leaves and in the second, less stringent, dataset of RNA-seq in roots are shown in S3 Table.

### Gene Ontology enrichment analysis

**Figure 3:**
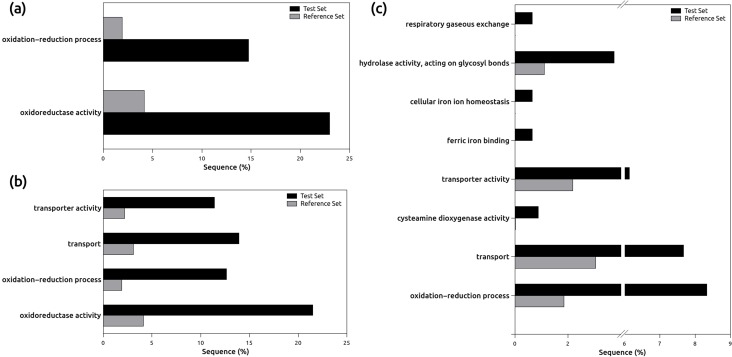
Gene Ontology enrichment analysis of the genes identified. (a) Significant GO terms (FDR *P*<0.05) over-represented among the up-regulated genes of the first, more stringent, dataset. (b) Significant GO terms (FDR *P*<0.05) over-represented among the DE genes of the first, more stringent, dataset. (c) Significant GO terms (FDR *P*<0.005) over-represented among the DE genes of the second, less stringent, dataset. Test Set is the set of the up-regulated genes, Reference Set is the background of the *P*. *trichocarpav 2*.*2* GO terms mapping.

Gene Ontology enrichment analysis [37] was used to identify important processes involved in excess Zn response in *P*. *x canadensis* clone I-214. Gene Ontology enrichment analysis of the first, more stringent, dataset showed a significant (FDR *P*<0.05) over-representation of GO-terms related to oxidoreductase activity (GO:0016491, 22.9%, FDR *P* = 3.7e^-4^) and oxidation-reduction process (GO:0055114, 14.75%, FDR *P* = 0.002) for the up-regulated genes (Fig. 3A); for the down-regulated genes there was no significant enrichment of GO terms. However, when considering together the up- and down-regulated genes identified in the first, more stringent, dataset also the GO terms related to transport (GO:0006810, 13.9%, FDR *P* = 0.016) and transporter activity (GO:0005215, 11.4%, FDR *P* = 0.02) (Fig. 3B, S4 Table) were significantly enriched. These last two GO categories include eight genes belonging to the up-regulated set (POPTR_0018s00450, POPTR_0005s03840, POPTR_0008s17960, POPTR_0009s03950, POPTR_0002s08090, POPTR_0017s11030, POPTR_0010s08250, POPTR_0002s08100) and three genes belonging to the down-regulated set (POPTR_0008s04860, POPTR_0008s08310, POPTR_0005s20350). By considering the functional annotations of the most similar *Arabidopsis* homologs, the up-regulated genes related to the GO term “transport” appear likely to be involved in metal uptake and detoxification, while the down-regulated genes appear more likely to be involved in exclusion and exocytosis (Table 2).

Gene Ontology enrichment analysis of the second, less stringent, dataset identified a similar pattern, with addition of several biologically sensible and logically related GO terms (Fig. 3c, S5 Table). Indeed, the more significant (FDR *P*<0.005) GO terms are oxidation-reduction process (GO:0055114, 8.3%, FDR *P* = 1.14e^-10^), cysteamine dioxygenase activity (GO:0047800, 0.8%, FDR *P* = 2.65e^-6^), transport (GO:0006810, 7.6%, FDR *P* = 3.12e^-4^), transporter activity (GO:0005215, 6.1%, FDR *P* = 3.12e^-4^), cellular iron ion homeostasis (GO:0006879, 0.66%, FDR *P* = 0.002), ferric iron binding (GO:0008199, 0.66%, FDR *P* = 0.002), respiratory gaseous exchange (GO:0007585, 0.66%, FDR *P* = 0.003) and hydrolase activity (GO:0004553, 3.5%, FDR *P* = 0.004).

## Discussion

Next Generation Sequencing technologies have become an invaluable tool for high-throughput differential expression analyses, but in non-model plants their use is still challenging due to the lack or incompleteness of reference genome sequences, mis-annotation [38] and high rates of gene duplications [39]. Although the reference genome for *P*. *trichocarpa* is available [12], differential expression analyses are complicated by the high intra-specific genetic variability and polyploidy of this species [30]. Validation by RT-qPCR, with an agreement >90% and a correlation coefficient (R) of 0.93 between the two experimental techniques, highlights the specificity of our approach at both qualitative and quantitative levels. This also suggests that our approach is reliable and could also be successfully applied to other hybrid poplar genotypes.

The comparison of growth parameters and Zn quantification in the aerial parts and in roots between control and treated plants of *P*. *x canadensis* clone I-214 are largely consistent with our previous studies based on similar experiments with the same *Populus* clone [18], [19], [20], [27]. These results confirm the phenotypic response of this clone towards Zn excess—anindicator phenotype—with internal Zn concentration increasing by the increase of external levels. The highest Zn concentration was revealed in the roots, followed by leaves and finally the stem, demonstrating a greater accumulation in the roots, but also a good ability of translocation to the leaves. This behavior is typical of indicator plants that does not exclude the metal but its uptake and accumulation are proportional to the metal level in the growth medium. However, the Zn uptake in I-214 is not so elevated to be classified as Zn accumulator or hyperaccumulator plant [7], [20], [24].

The low overlaps between the genes identified as DE in both the RNA-seq root analysis and the leaf microarray expression profiling, obtained from the same clone under the same Zn treatments [20], suggests different molecular responses between these organs towards excess Zn, also due to their different Zn concentration in these two organs. In the first, more stringent, dataset the over-representation of GO terms related to oxidation-reduction and oxidoreductase activity likely reflects a defensive strategy to the Zn-mediated production of Reactive Oxygen Species, probably mediated by glutathione conjugation [40]. The activation of the same mechanism was also reported in previous studies on leaves of this clone under excess Zn [18]-[20]. In the same stringent dataset, the up-regulation of genes related to the “transport” GO term, putatively involved in metal uptake and vacuolar detoxification, could be linked to the enhanced Zn uptake and sequestration in roots. In addition, the concurrent down-regulation of genes involved in metal efflux might reduce Zn movement to the aerial parts, consistent I-214 response to excess Zn which reveals a Zn tolerance but not a Zn hyperaccumulation capacity of this plant [7], [20]. This hypothesis would be in agreement with previous studies, which report vacuolar sequestration as a conserved mechanism used by plants and other organisms to detoxify excess Zn at the cellular level [41], [42]. This also confirms the observation that vacuolar metal uptake, mediated mainly by metal chelators and vacuolar transporters, occurs in poplar roots [23], [43].

The enrichment of GO terms related to oxidation-reduction process, transport and transporter activity in the less stringent dataset suggests that many genes, showing consistent if only marginal significance of differential expression, are likely involved in the same physiological processes as the genes from the more stringent set, underlining the relevance of such processes in *in viv*o responses to zinc excess. Despite this, the differential expression of these genes is based only on bioinformatic prediction. Therefore, for further discussion we consider only the genes in the first, more stringent, dataset whose differential expression was validated by RT-qPCR.

Among the genes related to the “transport” GO term, we observed the up-regulation of genes likely involved in metal chelation (POPTR_0005s03840), detoxification of glutathione-conjugated molecules (POPTR_0008s17960) [44], and metal uptake (POPTR_0009s03950, POPTR_0002s08100). The *Arabidopsis* orthologs of these last two—*ZRT/IRT-like protein 2* (POPTR_0009s03950) and *NRAMP1* (POPTR_0002s08100)—are well-studied genes involved in Fe and Zn homeostasis. *AtNRAMP1* is a member of the Natural Resistance Associated protein family. This gene encodes for a plasma membrane Fe and Mn transporter that is up-regulated in response to the deficiency of both these metals [45], [46]. When expressed under *IRT1* promoter in *Arabidopsis irt-1* loss-of-function mutant, *AtNRAMP1* gene partially restores iron uptake and increases also Mn, Co and Zn content compared to the untransformed mutants [46]. The ortholog gene from *Malus baccata* (*MbNRAMP1*) is able to complement yeast mutant strains defective for both low-/high-affinity iron and manganese uptake when expressed in yeast, and also to transport Cd^2+^ [47]. *NRAMP1* from *Oryza sativa* showed Cd-uptake activity when expressed in yeast, and its up-regulation in Cd-accumulating rice cultivar, together with the increased Cd uptake and sensitivity in over-expressing transgenic rice, suggest also an involvement in Cd homeostasis of this protein [48]. Since the *NRAMP1 Populus* ortholog is up-regulated in response to Zn excess in root, we suggest its possible involvement in Zn/Fe uptake and that it could be a candidate target for further studies and also for the possibly managing Zn homeostasis and uptake in *Populus* species.

*AtZRT/IRT-like protein 2*, known also as *AtZIP2*, belongs to the ZIP transporter family that is involved in the transport of metals including Zn, Cu, Mn and Fe [49–53]. The *Arabidopsis* genome encodes 15 ZIP transporters [54], the best characterized being IRT1 and IRT2 Fe and Zn transporters [55], reported to be up-regulated in response to both iron deficiency and excess Zn [56]. Interestingly, considering the seedling lethality of *irt-1 Arabidopsis* mutant [57] and the central role of this gene in regulating Fe/Zn uptake in this species [58], with a limited involvement of *IRT2* [53], no clear annotated orthologs of *IRT* genes are present in the *P*. *trichocarpa* genome. This could suggests the involvement of different genes in regulating metal homeostasis between these two species. Instead *AtZIP2* has been shown to be up-regulated under both Zn and iron deficiency, and only little up-regulated in response to a Zn surplus [53], [59], suggesting a marginal involvement of this gene in response to Zn excess in *Arabidopsis*. However, the strong up-regulation of poplar *ZRT/IRT-like protein 2* in the root of *P*. *x canadensis* clone I-214 exposed to excess Zn suggests a putative role for this gene in regulating Zn homeostasis also in this species.

The interaction between Fe and Zn homeostasis mechanisms has been widely reported in *Arabidopsis* [56], [59], and we can hypothesize that similar interactions might also occur in poplar. In *Arabidopsis* the principal transcription factor involved in response to Fe-deficiency in roots is *FIT* (FER-like-iron-deficiency-induced transcription factor) [60], which increases the expression of genes such as *IRT1* and *IRT2* involved in Fe/Zn uptake. A recent study reports also its up-regulation in response to excess Zn and Cd [61], [62]. In our experiments the *P*. *trichocarpa* ortholog of *FIT* (POPTR_0009s01100) was down-regulated ~5 fold in response to excess Zn. This results suggests divergent mechanisms of transcriptional regulation in response to excess Zn, and distinct interactions within Zn/Fe homeostasis networks in poplar.

Interestingly, a recent comparison of transcriptome responses under excess Zn between *Arabidopsis thaliana* and its closest related hyperaccumulator *Arabidopsis halleri* showed that *FIT* and members of the ZIP family were DE in the hyperaccumulator plants, consistent with a meaningful role for these genes in the enhanced Zn tolerance and accumulation in *A*. *halleri* [61], [63]. Accordingly, we suggest that the *Populus* orthologs of *ZRT/IRT-protein like 2* and *FIT* (POPTR_0009s03950, POPTR_0009s01100), identified as DE in our analysis, could be involved in the regulation of Zn homeostasis in poplar, and possibly they could be useful candidates for manipulating Zn tolerance in this plant.

Despite the contrasting behavior of FIT in response to excess Zn between *Arabidopsis* and *Populus*, the differential expression of other genes, whose closest *Arabidopsis* homologs are involved in or related to the regulation of both Fe/Zn homeostasis, is consistent with conserved function in poplar. This hypothesis is underlined by the enrichment of GO terms related to cellular iron ion homeostasis (GO:0006879) and ferric iron ion binding (GO:0008199) in the set of DE genes identified under less stringent statistical parameters, highlighting also the utility of extracting two different sets of candidate genes.

Up-regulated genes putatively involved in regulation of Fe/Zn homeostasis included: POPTR_0006s03590 and POPTR_0016s03680, two apparent orthologs of *Arabidopsis bHLH38*, and POPTR_0012s05140, an ortholog of *BTS*. *AtbHLH38* encodes a transcription factor up-regulated both under excess Zn and Fe-deficiency in a *FIT* independent manner [64], consistent with a conserved role for this transcription factor. Instead *AtBTS* encodes for a poorly characterized zinc finger containing protein involved in a distinct Fe-deficiency responsive network from *FIT* [65], [66], and acts by negatively regulating this stress response [67]. The up-regulation of its putative *Populus* ortholog POPTR_0012s05140 points to possible additional roles in Zn-stress responses and is, to the best of our knowledge, the first information regarding this gene in poplar.

Among other genes related to metal homeostasis and mobilization, POPTR_0004s20440—annotated as the ortholog of *NAS4* in *Arabidopsis -* is down-regulated. Nicotianamine (NA), synthetized by NAS genes, is a non-proteinogenic amino acid involved in intracellular metal chelation and long-distance phloem metal transport [68]. In *Arabidopsis thaliana* NAS genes were up-regulated in response to Fe-, Zn- and Cu-deficiency [51], [69]. Recent work using an *Arabidopsis halleri* NAS2-knockdown mutant highlights the key role of this protein in the hyperaccumulator phenotype of this plant [70]. Furthermore, studies using transgenic tobacco plants also indicate that NA was involved in long-distance transport of Zn [71], while rice plants over-expressing NAS genes are more resistant to Fe and Zn deficiency and less tolerant to excess Zn, Cu and Ni than wild type plants [72]. In this light, we interpret the down-regulation of POPTR_0004s20440 in response to excess Zn as a mechanism for reducing Zn mobility from roots to aerial parts, a common physiological response of this clone towards Zn stress [19],[20],[27]. Even though several studies report that *HMA4* plays a central role in increasing metal tolerance and accumulation in herbaceous species [73], [74], these mechanisms seem not to be conserved in *Populus* species. Indeed, *Populus trichocarpa HMA4* was not significantly differentially expressed in response to similar Zn concentration in poplar roots [24], suggesting that the reduced Zn translocation observed in this species could be mediated by different molecular mechanisms. Instead the nicotiamine-mediated metal transport seems to be more conserved among different species and we suggest that the modulation of expression of this gene might represent a plausible strategy for enhancing metal translocation and accumulation in aerial parts in poplar.

Overall, our results suggest that, besides activating oxidative-stress responses, excess Zn also modulates mechanisms involved in Zn-induced Fe-deficiency by altering the expression of transcription factors, up-regulating genes involved in metal uptake and down-regulating genes involved in metal translocation. In particular, we observed that the genes regulating the Zn-induced Fe deficiency response behave differently between *Populus* and *Arabidopsis thaliana*. In *Arabidopsis thaliana* excess Zn reduces shoot Fe content and induced the expression of *FIT*, *IRT1* and *IRT2* [61], [75]. The activation of these transporters could be a mechanism to cope with the Zn-induced Fe-deficiency, but could be also responsible of the Zn sensitivity of *Arabidopsis thaliana*. Since *IRT1* and *IRT2* can transport also Zn, they probably overload the sub-cellular detoxification mechanisms of *A*. *thaliana* [75]. On the other hand, the hyperaccumulator *A*. *halleri* showed a general lower expression of these genes and Zn excess does not affect shoots Fe accumulation [61]. A previous study on the same *Populus* I-214 clone (and the same exact experimental setup) did not show any reduction in Fe concentration in leaves under excess Zn [20]. This could suggests that under excess Zn *Populus* avoid Zn-induced Fe deficiency by silencing the *FIT* transcriptional network. The down-regulation of *FIT* could reduce a possible aspecific uptake of excessive Zn from the roots, like observed in *Arabidopsis thaliana*. On the other hand, the Fe homeostasis could be maintained by the activation of FIT-independent transcriptional networks, probably regulated by the *Populus* homologous of the two *bHLH* transcription factor and *BTS* identified as DE in our experiment. These findings constitute a starting point for understanding Zn homeostasis in poplar plants, possibly manipulating metal-stress responses of these plants, but also for improving the phytoremediation performances of poplar. However, we have to consider that our experiment, as most of those conducted on the molecular biology of plant roots (e.g.: [21], [24], [55], [62]) was carried out in a growth chamber in nutrient solution. These conditions exclude variability due to weather events, but above all reduce the enormous complexity of the rhizosphere that in “outdoor” soil conditions is created at the interface between roots and soil. Indeed, it has been shown that rhizosphere microorganisms, especially mycorrhizal fungi and bacteria, are able to increase the tolerance of plants against soil pollutants, by stimulating plant growth and contributing in this way to an accelerated remediation of contaminated soils [76], [77]. For these reasons, plants selected and/or transformed for molecular functions identified here for Zn tolerance will be tested in outdoor systems before involvement in phytoremediation applications.

In summary, NGS-based whole transcriptome analysis of Zn-stressed poplar roots, in conjunction with prior knowledge from *Arabidopsis* and other species, has allowed the identification of putative molecular mechanisms strongly consistent with the observed phenotypes and physiological responses. Both in the leaves and roots of poplar subjected to Zn excess the transcriptome analysis reveals the activation of defense systems against oxidative stress and alterations of the basic metabolism, even if in a diversified manner. Additional detailed functional analyses of individual genes highlighted in the current work and investigated in similar experiments and in soil growth systems will provide more precise insight into the molecular mechanisms underlying Zn stress responses in poplar, eventually facilitating strategies to improve woody plant tolerance to heavy metals and their phytoremediation performance.

## Supporting Information

S1 Table Expression of the DE genes. Normalized log_2_ Fold Change from read counts of *P*. *trichocarpa* orthologs of genes identified as DE in the second, less stringent, dataset.
(XLS)

S2 Table Primer list. List of primers used for validation of DE genes by RT-qPCR, with respective *P*. *trichocarpa* gene IDs
(XLS)

S3 Table Common up- and down-regulated genes in both leaves and roots. Annotation of the genes identified as up- or down-regulated in response to excess Zn in both leaf microarray analysis and in the second, less stringent dataset identified by root RNA-seq approach.
(XLS)

S4 Table Enriched Gene Ontology (GO) terms in the first, more stringent, dataset. List of the the enriched GO terms in the first, more stringent, dataset together with the respective *p values*.
(XLS)

S5 Table Enriched Gene Ontology (GO) terms in the second, less stringent, dataset. List of the enriched GO terms in the second, less stringent, dataset together with the respective *p values*.
(XLS)

## References

[1] Andreini C, Banci L, Bertini I, Rosato A (2006) Zinc through the three domains of life. J Proteome Res 5: 3173–3178. 17081069 10.1021/pr0603699

[2] Marschner H (1995) Mineral nutrition of higher plants, 2nd Ed. London, UK: Academic Press

[3] Broadley MR, White PJ, Hammond JP, Zelko I, Lux A (2007) Zinc in plants. New Phytol 173: 677–702. 17286818 10.1111/j.1469-8137.2007.01996.x

[4] Lado LR, Hengl T, Reuter HI (2008) Heavy metals in European soils: a geostatistical analysis of the FOREGS Geochemical database. Geoderma 148: 189–199.

[5] Vassilev A, Schwitzguebel JP, Thewys T, Van Der Lelie D, Vangronsveld J (2004) The use of plants for remediation of metal contaminated soils. ScientificWorldJournal 4: 9–34. 14755099 10.1100/tsw.2004.2 PMC5956303

[6] Frérot H, Faucon MP, Willems G, Godé C, Courseaux A, et al. (2010) Genetic architecture of zinc hyperaccumulation in *Arabidopsis halleri*: the essential role of QTL x environment interactions. New Phytol 187: 355–367. 10.1111/j.1469-8137.2010.03295.x 20487314

[7] Maestri E, Marmiroli M, Visioli G, Marmiroli N (2010) Metal tolerance and hyperaccumulation: costs and trade-offs between traits and environment. Environ Exp Bot 68: 1–13.

[8] Sebastiani L, Scebba F, Tognetti R (2004) Heavy metal accumulation and growth responses in poplar clones Eridano (*Populus deltoides* × *maximowiczii*) and I-214 (*P*. × *euramericana*) exposed to industrial waste. Environ Exp Bot 52: 79–88.

[9] Laureysens I, De Temmerman L, Hasti T, Van Gysel M, Ceulemans R (2005) Clonal variation in heavy metal accumulation and biomass production in a poplar coppice culture: II. Vertical distribution and phytoextraction potential. Environ Pollut 133: 541–551. 15519729 10.1016/j.envpol.2004.06.013

[10] Pilon-Smits E (2005) Phytoremediation. Annu Rev Plant Biol 56: 15–39. 15862088 10.1146/annurev.arplant.56.032604.144214

[11] Pulford ID, Dickinson NM (2005) Phytoremediation technologies using trees In: Prasad MNV, Sajwan Kenneth S, Naidu R, editors. Trace Element in the Environment. Biogeochemistry, Biotechnology, and Bioremediation. Boca Raton, Florida, USA: Taylor and Francis pp. 383–403.

[12] Tuskan GA, Difazio S, Jansson S, Bohlmann J, Grigoriev I, et al (2006) The genome of black cottonwood, *Populus trichocarpa* (Torr. & Gray). Science 313: 1596–1604. 16973872 10.1126/science.1128691

[13] Schrader J, Nilsson J, Mellerowicz E, Berglund A, Nilsson P, et al (2004) A high resolution transcript profile across the wood-forming meristem of poplar identifies potential regulators of cambial stem cell identity. Plant Cell 16: 2278–2292. 15316113 10.1105/tpc.104.024190 PMC520933

[14] Dharmawardhana P, Brunner AM, Strauss SH (2010) Genome-wide transcriptome analysis of the transition from primary to secondary stem development in *Populus trichocarpa* . BMC Genomics 11: 150 10.1186/1471-2164-11-150 20199690 PMC2846914

[15] Ralph SG, Chun HJ, Cooper D, Kirkpatrik R, Kolosova N, et al (2008) Analysis of 4,664 high-quality sequence-finished poplar full-length cDNA clones and their utility for the discovery of genes responding to insect feeding. BMC Genomics 9: 57 10.1186/1471-2164-9-57 18230180 PMC2270264

[16] Berta M, Giovannelli A, Sebastiani F, Camussi A, Racchi ML (2010) Transcriptome changes in the cambial region of poplar (*Populus alba* L.) in response to water deficit. Plant Biol 12: 341–354. 10.1111/j.1438-8677.2009.00320.x 20398240

[17] Chen S, Jiang J, Li H, Liu G (2012) The salt-responsive transcriptome of *Populus simonii x Populus nigra* via DGE. Gene 504: 203–212. 10.1016/j.gene.2012.05.023 22634611

[18] Di Baccio D, Kopriva S, Sebastiani L, Rennenberg H (2005) Does glutathione metabolism have a role in the defence of poplar against zinc excess? New Phytol 167: 73–80. 15948831 10.1111/j.1469-8137.2005.01462.x

[19] Di Baccio D, Tognetti R, Minnocci A, Sebastiani L (2009) Responses of the *Populus x euramericana* clone I.214 to excess zinc: carbon assimilation, structural modification, metal distribution and cellular localization. Environ Exp Bot 67:153–163.

[20] Di Baccio D, Galla G, Bracci T, Andreucci A, Barcaccia G, et al (2011) Transcriptome analyses of *Populus x euramericana* clone I-214 leaves exposed to excess zinc. Tree Physiol 31: 1293–1308. 10.1093/treephys/tpr106 22038866

[21] Grisel N, Zoller S, Künzli-Gontarczyk M, Lampart T, Münsterkötter M, et al (2010) Transcriptome responses to aluminum stress in roots of aspen (*Populus tremula*). BMC Plant Biol 10: 185 10.1186/1471-2229-10-185 20727216 PMC3017830

[22] He J, Li H, Luo J, Ma C, Li S, et al (2013) A transcriptomic networks underlies microstructural and physiological responses to cadmium in *Populus x canescens* . Plant Physiol 162: 424–439. 10.1104/pp.113.215681 23530184 PMC3641221

[23] Blaudez D, Kohler A, Martin F, Sanders D, Chalot M (2003) Poplar metal tolerance protein 1 confers zinc tolerance and is an oligomeric vacuolar zinc transporter with an essential leucine zipper motif. Plant Cell 15: 2911–2928. 14630973 10.1105/tpc.017541 PMC282827

[24] Adams JP, Adeli A, Hsu CY, Harkess RL, Page GP, et al (2011) Poplar maintains zinc homeostasis with heavy metal genes HMA4 and PCS1. J Exp Bot 62: 3737–3752. 10.1093/jxb/err025 21504875 PMC3134336

[25] Kholer A, Delaruelle C, Martin D, Encelot N, Martin F (2003) The poplar root transcriptome: analysis of 7000 expressed sequence tags. FEBS Lett 542: 37–41. 12729894 10.1016/s0014-5793(03)00334-x

[26] Baker AJM (1981) Accumulatora and excluders—strategies in the response of plants to heavy metals. J Plant Nutr 3: 643–654.

[27] Stoláriková M, Vaculík M, Lux A, Di Baccio D, Minnocci A, et al (2012) Anatomical differences in poplar (*Populus x euramericana* clone I-214) roots exposed to zinc excess. Biologia 67: 483–489.

[28] Lux A, Martinka M, Vaculík M, White PJ (2011) Root responses to cadmium in the rhizosphere: a review. J Exp Bot 62: 21–37. 10.1093/jxb/erq281 20855455

[29] Ozsolak F, Milos PM (2011) RNA sequencing: advances, challenges and opportunities. Nat Rev Genet 12: 87–98. 10.1038/nrg2934 21191423 PMC3031867

[30] Stanton BJ, Neale DB, Li S (2010) *Populus* breeding: from the classical to the genomic approach In: Jansson S, Bhalerao RP, Groover AT, editors. Genetics and genomics of Populus, Plant genetic and genomics: crops and model. New York, USA Springer Press pp 309–348.

[31] Di Baccio D, Tognetti R, Sebastiani L, Vitagliano C (2003) Responses of *Populus deltoides x Populus nigra* (*Populus x euramericana*) clone I-214 to high zinc concentration. New Phytol 159: 443–452. 10.1046/j.1469-8137.2003.00818.x 33873348

[32] Li R, Yu C, Li Y, Lam TW, Yiu SM, et al (2009) SOAP2: an improved ultrafast tool for short read alignment. Bioinformatics 25: 1966–1967. 10.1093/bioinformatics/btp336 19497933

[33] Anders S, Huber W (2010) Differential expression analysis for sequence count data. Genome Biol 11: R106 10.1186/gb-2010-11-10-r106 20979621 PMC3218662

[34] Wu Z, Jenkins BD, Rynearson TA, Dyhrman ST, Sito MA, et al (2010) Empirical bayes analysis of sequencing-based transcriptional profiling without replicates. BMC Bioinformatics 11: 564 10.1186/1471-2105-11-564 21080965 PMC3098101

[35] Brunner AM, Yakovlev IA, Strauss SH (2004) Validating internal controls for quantitative plant gene expression studies. BMC Plant Biol 4: 14 15317655 10.1186/1471-2229-4-14 PMC515301

[36] Conesa A, Götz S, Garcia-Gómez JM, Terol J, Talón M, et al (2005) Blast2GO: a universal tool for annotation, visualization and analysis in functional genomics research. Bioinformatics 21: 3674–3676. 16081474 10.1093/bioinformatics/bti610

[37] Blütghen N, Brand K, Cajavec B, Swat M, Herzel H, et al (2005) Biological profiling of gene groups utilizing gene ontology. Genome Inform 16: 106–115. 16362912

[38] Bräutigam A, Gowik U (2010) What can next generation sequencing do for you? Next generation sequencing as a valuable tool in plant research. Plant Biol 12: 831–841. 10.1111/j.1438-8677.2010.00373.x 21040298

[39] Severing EI, Van Dijk ADJ, Stiekema WJ, Van Ham RCHJ (2009) Comparative analysis indicates that alternative splicing in plants has a limited role in functional expansion of the proteome. BMC Genomics 10: 154 10.1186/1471-2164-10-154 19358722 PMC2674458

[40] Mithöfer A, Schulze B, Boland W (2004) Biotic and heavy metal stress response in plant: evidence for common signals. FEBS Lett 566: 1–5. 15147858 10.1016/j.febslet.2004.04.011

[41] Morel M, Crouzet J, Gravot A, Auroy P, Leonhardt N, et al (2009) AtHMA3, a P1B-ATPase allowing Cd/Zn/Co/Pb vacuolar storage in *Arabidopsis* . Plant Physiol 149: 894–904. 10.1104/pp.108.130294 19036834 PMC2633814

[42] Potters G, Pasternak TP, Guisez Y, Jansen MK (2009) Different stresses, similar morphogenic response: integrated a plethora of pathways. Plant Cell Environ 32: 158–169. 10.1111/j.1365-3040.2008.01908.x 19021890

[43] Desbrosses-Fonrounge AG, Voigt K, Schröder A, Arrivault S, Thomine S, et al (2005) *Arabidopsis thaliana* MTP1 is a Zn transporter in the vacuolar membrane which mediate Zn detoxification and drives leaf Zn accumulation. FEBS Lett 579: 4165–4174. 16038907 10.1016/j.febslet.2005.06.046

[44] Martinoia E, Klein M, Geisler M, Bovet L, Forestier C, et al (2002) Multifunctionality of plant ABC transporters-more than just detoxifiers. Planta 214: 345–355. 11855639 10.1007/s004250100661

[45] Curie C, Alonso JM, Le Jean M, Ecker JR, Briat JF (2000) Involvement of NRAMP1 from *Arabidopsis thaliana* in iron transport. Biochem J 347: 749–755. 10769179 PMC1221012

[46] Cailliatte R, Schikora A, Briat JF, Mari S, Curie C (2010) High-affinity manganese uptake by the metal transporter NRAMP1 is essential for *Arabidopsis* growth in low manganese condition. Plant Cell 22: 904–917. 10.1105/tpc.109.073023 20228245 PMC2861449

[47] Xiao H, Yin L, Xu X, Han Z (2008) The iron-regulated transporter, MbNRAMP1, isolated from *Malus baccata* is involved in Fe, Mn and Cd trafficking. Ann Bot 102: 881–889. 10.1093/aob/mcn178 18819951 PMC2712396

[48] Takahashi R, Ishimau Y, Senoura T, Shimo H, Ishikawa S, et al (2011) The OsNRAMP1 iron transporter is involved in Cd accumulation in rice. J Exp Bot 62: 4843–4850. 10.1093/jxb/err136 21697258 PMC3192999

[49] Eide D, Broderius M, Fett J, Guerinot ML (1996) A novel iron-regulated metal transporter from plants identified by functional expression in yeast. Proc Natl Acad Sci USA 93: 5624–5628. 8643627 10.1073/pnas.93.11.5624 PMC39298

[50] Grotz N, Fox T, Connolly E, Park W, Guerinot ML, et al (1998) Identification of a family of zinc transporter genes from *Arabidopsis* that respond to zinc deficiency. Proc Natl Acad Sci USA 95: 7220–7224. 9618566 10.1073/pnas.95.12.7220 PMC22785

[51] Wintz H, Fox T, Wu YY, Feng V, Chen W, et al (2003) Expression profiles of *Arabidopsis thaliana* in mineral deficiencies reveal novel transporters involved in metal homeostasis. J Biol Chem 278: 47644–47653. 13129917 10.1074/jbc.M309338200

[52] Pedas P, Ytting CK, Funglsang AT, Jahn TP, Schjoerring JK, et al (2008) Maganese efficiency in barley: identification and characterization of the metal ion transporter HvIRT. Plant Physiol 148: 455–466. 10.1104/pp.108.118851 18614714 PMC2528110

[53] Milner MJ, Seamon J, Craft E, Kochian LV (2013) Transport properties of members of the ZIP family in plants and their role in Zn and Mn homeostasis. J Exp Bot 64: 369–381. 10.1093/jxb/ers315 23264639 PMC3528025

[54] Colangelo EP, Guerinot ML (2006) Put the metal to the petal: metal uptake and transport throughout plants. Curr Opin Plant Biol 9: 322–330. 16616607 10.1016/j.pbi.2006.03.015

[55] Vert G, Barberon M, Zelazny E, Séguéla M, Briat JF, et al (2009) *Arabidopsis* IRT2 cooperates with the high-affinity iron uptake system to maintain iron homeostasis in root epidermal cells. Planta 229: 1171–1179. 10.1007/s00425-009-0904-8 19252923

[56] Sinclair SA, Krämer U (2012) The zinc homeostasis network of land plants. Biochim Biophys Acta 1823: 1553–1567. 10.1016/j.bbamcr.2012.05.016 22626733

[57] Henriques R, Jásik J, Klein M, Martinoia E, Feller U, et al (2002) Knock-out of *Arabidopsis* metal transporter gene *IRT1* results in iron deficiency accompanied by cell differentiation defects. Plant Mol Biol 50: 587–597. 12374293 10.1023/a:1019942200164

[58] Vert G (2002) IRT1, an *Arabidopsis* transporter essential for iron uptake from the soil and for plant growth. Plant Cell 14: 1223–1233. 12084823 10.1105/tpc.001388 PMC150776

[59] Yang JWY, Lin WD, Schmidt W (2010) Transcriptional profiling of the *Arabidopsis* iron deficiency response reveals conserved transition metal homeostasis networks. Plant Physiol 152: 2130–2141. 10.1104/pp.109.152728 20181752 PMC2850031

[60] Yuan YX, Zhang J, Wang DW, Ling HQ (2005) AtbHLH29 of *Arabidopsis thaliana* is a functional ortholog of tomato FER involved in controlling iron acquisition in Strategy I plants. Cell Res 15: 613–62. 16117851 10.1038/sj.cr.7290331

[61] Shanmugam V, Lo JC, Wu CL, Wang SL, Lai CC, et al (2011) Differential expression and regulation of iron-regulated metal transporter in *Arabidopsis halleri* and *Arabidopsis thaliana*- the role in zinc tolerance. New Phytol 190: 125–137. 10.1111/j.1469-8137.2010.03606.x 21219335

[62] Wu H, Chen C, Du J, Liu H, Cui Y, et al (2012) Co-overexpression *FIT* with *AtbHLH38* or *AtbHLH39* in *Arabidopsis*-enhanced cadmium tolerance via increased cadmium sequestration in roots and improved iron homeostasis of shoots. Plant Physiol 158: 790–800. 10.1104/pp.111.190983 22184655 PMC3271767

[63] Weber M, Harada E, Vess C, Roepenack-Lahaye E, Clemens S (2004) Comparative microarray analysis of *Arabidopsis thaliana* and *Arabidopsis halleri* roots identifies nicotianamine synthase, a ZIP transporter and other genes as potential metal hyperaccumulation factors. Plant J 37: 269–281. 14690510 10.1046/j.1365-313x.2003.01960.x

[64] Wang HY, Klatte M, Jakoby M, Bäumlein H, Weisshaar B, et al (2007) Iron deficiency-mediated stress regulation of four subgroup Ib BHLH genes in *Arabidopsis thaliana* . Planta 226: 897–908. 17516080 10.1007/s00425-007-0535-x

[65] Hindt MN, Guerinot ML (2012) Getting a sense of signals: Regulation of the plant iron deficiency response. Biochim Biophys Acta 1823: 1521–1530. 10.1016/j.bbamcr.2012.03.010 22483849 PMC4008143

[66] Kobayashi T, Nagasaka S, Senoura T, Itai RN, Nakanishi H, et al (2013) Iron-binding haemerythrin RING ubiquitin ligases regulate plant iron responses and accumulation. Nat Commun 4: 2792 10.1038/ncomms3792 24253678 PMC3905729

[67] Long AT, Tsukagoshi H, Busch W, Lahner B, Salt DE, et al (2010) The bHLH transcription factor POPEYE regulates response to iron deficiency in *Arabidopsis* roots. Plant Cell 22: 2219–2236. 10.1105/tpc.110.074096 20675571 PMC2929094

[68] Curie C, Briat JF (2003) Iron transport and signaling in plants. Annu Rev Plant Biol 54: 183–206. 14509968 10.1146/annurev.arplant.54.031902.135018

[69] Klatte M, Schuler M, Wirtz M, Fink-Straube C, Hell R, et al (2009) The analysis of *Arabidopsis* nicotianamine synthase mutants reveals functions for nicotianamine in seed iron loading and iron deficiency responses. Plant Physiol 150: 257–271. 10.1104/pp.109.136374 19304929 PMC2675739

[70] Deinlein U, Weber M, Schmidt H, Rensch S, Trampczynska A, et al (2012) Elevated nicotianamine levels in *Arabidopsis halleri* roots play a key role in zinc hyperaccumulation. Plant Cell 24: 708–723. 10.1105/tpc.111.095000 22374395 PMC3315242

[71] Takahashi M, Terada Y, Nakai I, Nakanishi H, Yoshimure E, et al (2003) Role of nicotianamine in the intracellular delivery of metal and plant reproductive development. Plant Cell 15: 1263–1280. 12782722 10.1105/tpc.010256 PMC156365

[72] Lee S, Jeon US, Lee SJ, Kim YK, Persson DP, et al (2009) Iron fortification of rice seeds through activation of the nicotianamine synthase gene. Proc Natl Acad Sci USA 106: 22014–22019. 10.1073/pnas.0910950106 20080803 PMC2799860

[73] Hanikenne M, Talke IN, Haydon MJ, Lanz C, Nolte A, et al (2008) Evolution of metal hyperaccumulation required cis-regulatory changes and triplication of *HMA4* . Nature 453: 391–395. 10.1038/nature06877 18425111

[74] Cracium AR, Meyer CL, Chen J, Roosens N, De Groodt R, et al (2012) Variation in HMA4 gene copy number and expression among *Noccaea caerulescens* population presenting different levels of Cd tolerance and accumulation. J Exp Bot 63: 4179–4189. 10.1093/jxb/ers104 22581842

[75] Shanmugam V, Lo JC, Yeh KC (2013) Control of Zn uptake in Arabidopsis halleri: a balance between Zn and Fe. Front Plant Sci 4: 218 10.3389/fpls.2013.00218 23966999 PMC3744811

[76] Erland S, Taylor FS (2002) Diversity of ecto-mycorrhizal fungal communities in relation to the abiotic environment In: van der Heijden MGA, Sanders IR (eds) Mycorrhizal ecology. Springer, Berlin, Heidelberg, New York, pp 163–200

[77] Schützendübel A, Polle A (2002) Plant responses to abiotic stresses: heavy metal-induced oxidative stress and protection by mycorrhization. J Exp Bot 53:1351–1365. 11997381

